# “Awake and Ablated”: First experience with a balloon-in-basket PFA system without propofol

**DOI:** 10.1016/j.hroo.2025.11.011

**Published:** 2025-11-21

**Authors:** Roland Richard Tilz, Jan-Per Wenzel, Charlotte Eitel, Sorin Popescu, Julius Nikorowitsch, Karl-Heinz Kuck, Sascha Hatahet

**Affiliations:** 1 University Heart Center Lübeck, Department of Rhythmology, University Hospital Schleswig-Holstein, Campus Lübeck, Germany; 2 German Center for Cardiovascular Research (DZHK), Partner Site Hamburg/Kiel/Lübeck, Lübeck, Germany

**Keywords:** Pulmonary vein isolation, Pulsed field ablation, Conscious sedation, Atrial fibrillation, Patient-reported outcomes, Balloon-in-basket catheter

## Abstract

**Background:**

Pulsed field ablation (PFA) is a novel technique for pulmonary vein isolation (PVI) in atrial fibrillation, offering myocardial selectivity and a favorable safety profile. The balloon-in-basket (BiB)-PFA system enables circumferential PVI with optimized tissue contact. PFA is typically performed under deep sedation or general anesthesia, increasing procedural risk and logistical burden.

**Objective:**

This study evaluated the safety, feasibility, and patient experience of BiB-PVI under conscious sedation, keeping patients awake and avoiding routine use of propofol.

**Methods:**

Consecutive patients undergoing de novo PVI were prospectively enrolled. Conscious sedation included fentanyl, midazolam, metamizole, and lidocaine; propofol was used for direct-current cardioversion only. Patient experience was assessed via structured questionnaires at 3 time points: during ablation and 1 hour and 1 day after the procedure. Pain, dyspnea, and anxiety were rated on numeric rating scales (0–10); satisfaction and recommendation were rated on visual analog scales (0%–100%).

**Results:**

14 patients (86% male; age 70.6 ± 9.8 years; BMI 27.7 ± 3.7 kg/m^2^) were included. Procedure duration was 54.5 minutes (38.3, 60.8); LA dwell time was 23 minutes (19.5, 34.8). 2.5 PFA applications (2.0, 4.0) were delivered per vein (left superior pulmonary vein 2.5; left inferior pulmonary vein 2.5; right inferior pulmonary vein 2.5; right superior pulmonary vein 2.5). Additional ablation strategies were performed owing to the interventionist’s preference. No escalation to deep sedation was required. Pain was 6.5 (4.3, 8.0), decreasing to 2.0 after ablation and on day 1 (*P* = .002/.001). Dyspnea and anxiety remained low. Satisfaction reached 95% (90, 100) and recommendation 100% (90, 100), remaining high throughout follow-up.

**Conclusion:**

PFA using the BiB-PFA system under conscious sedation is safe, feasible, and well tolerated, enabling streamlined workflows with high patient satisfaction.

## Graphical abstract

**Figure:**
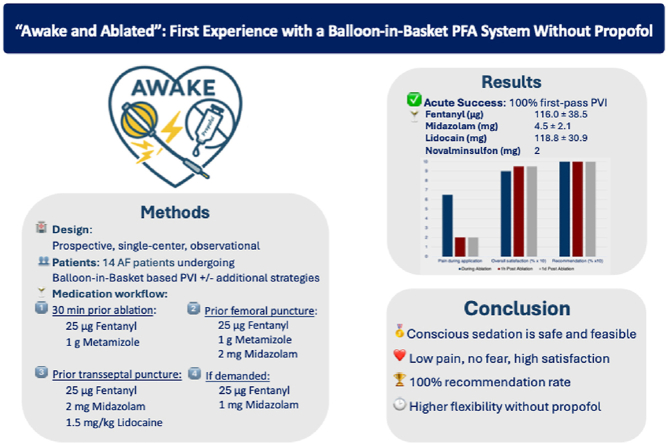

**Key Findings**

▪ Pulmonary vein isolation using balloon-in-basket pulsed field ablation was performed safely and feasibly under multimodal conscious sedation (fentanyl, midazolam, metamizole, and lidocaine) without the need for escalation to deep sedation.
▪ Patients reported high comfort and satisfaction levels, as well as a high likelihood of recommending the procedure, based on structured and time-resolved follow-up assessments.
▪ This approach may enhance procedural autonomy, improve resource efficiency, and support patient-centered care in routine electrophysiology practice.

## Introduction

Pulsed field ablation (PFA) has rapidly emerged as a transformative modality for pulmonary vein isolation (PVI) in patients with atrial fibrillation (AF).^1^ Unlike thermal ablation, PFA applies high-voltage electric fields to induce irreversible electroporation, enabling tissue-selective myocardial ablation while minimizing collateral damage, including phrenic nerve or esophageal injury and pulmonary vein stenosis.^2^, ^3^, ^4^

The VOLT system is a novel balloon-in-basket (BiB)-PFA catheter that integrates a compliant balloon with an expandable multielectrode lattice to ensure stable positioning and reliable electrode–tissue contact. Its design enables homogeneous circumferential energy delivery and enhanced procedural control. The system features contact-sensing capabilities compatible with 3-dimensional electroanatomic mapping. Initial clinical experience has demonstrated effective PVI with promising procedural efficiency, safety, and lesion durability.^5^^,^^6^ Nevertheless, most PFA procedures to date, including those performed with the BiB-PFA system, have been conducted under general anesthesia or deep sedation.^7^, ^8^, ^9^, ^10^ Although this approach ensures patient immobility and procedural control, it also entails risks such as respiratory depression, hemodynamic instability, and prolonged recovery—particularly in older or multimorbid patients.^11^ In addition, in many centers, propofol administration is restricted to anesthesiologists, limiting procedural flexibility and increasing resource requirements.^10^

Thereby, the BiB design may offer unique advantages. With stable wall contact and controlled energy delivery, it may elicit fewer extracardiac muscle contractions than other PFA platforms. This procedural stability and reduced neuromuscular stimulation could lower patient discomfort, enabling ablation under conscious rather than deep sedation. Such an approach may reduce sedation-related complications, simplify logistics, and allow real-time intraprocedural communication with the patient.

In this prospective study, we investigated the feasibility, safety, and patient-reported experience of PVI using the BiB-PFA system under a conscious sedation protocol without propofol.

## Methods

### Study population and trial design

This prospective, single-center study assessed the safety and feasibility of PVI with the BiB-PFA system under conscious sedation, enabling real-time evaluation of procedural tolerance and patient experience. From May 2025 to August 2025, consecutive patients with AF undergoing first-do PVI using the BiB-catheter (VOLT, Abbott) were enrolled.

Sedation level—conscious vs deep—was determined primarily by patient preference after structured counseling. None of the patients experienced anxiety or panic disorder. All participants were included in the Lübeck Ablation Registry. The study was approved by the local ethics committee (WF-028/15) and conducted in accordance with the Declaration of Helsinki.^12^ Eligible patients were ≥18 years old, with paroxysmal or persistent AF, and had provided a written consent.

### General procedural management

Vascular access was obtained via 2 ultrasound-guided femoral vein punctures (8F sheaths) after local anesthesia with 10 mL 2% xylocaine. A diagnostic catheter was positioned in the coronary sinus. Transseptal puncture was performed under fluoroscopy using a modified Brockenbrough technique and SL1 sheath (Abbott). Unfractionated heparin was administered after the puncture to maintain an activated clotting time of >300 seconds. The SL1 sheath was exchanged for a steerable 13 Fr sheath (Agilis NxT, Abbott). Pulmonary vein access was obtained over a 0.035" guidewire. To prevent vagal reactions, 1 mg intravenous (IV) atropine was given before ablation. PFA was delivered at 1800 V with ≥2 rotated applications/vein (maximum 8). Phrenic nerve capture was assessed via spline pacing in right-sided veins; if present, ablation proceeded at 1400 V with ≥3 applications. When areas of posterior or anterior fibrosis were identified via electroanatomic mapping using the BiB-PFA system, targeted ablation was performed with deselection of noncontacting splines. The number of applications and the choice of normal or reduced energy settings were at the operator’s discretion. Bidirectional conduction block was confirmed by demonstrating complete entrance and exit block across the respective lesion lines. In patients with concomitant typical atrial flutter, cavotricuspid isthmus ablation was performed with subsequent confirmation of bidirectional block by differential pacing. In the case of superior vena cava ablation, contrast injection was followed by a single test application train, and if no influence on sinus node function was observed, full application trains were delivered while the catheter was repositioned. Remapping was performed after ablation using the BiB-PFA system.

### Mild-sedation approach

The conscious sedation workflow, as presented in the manuscript, was developed stepwise during previous cases with a focus on medication timing and dosing, patient communication, and minimization of environmental stressors. All procedures followed a standardized institutional analgesia protocol tailored for awake ablation with the BiB-PFA system. Premedication began 15–30 minutes before the procedure with IV metamizole (1 g) and fentanyl (25 μg). Upon arrival in the electrophysiology (EP) laboratory, patients received an additional 1 g metamizole, 25 μg fentanyl, and 2 mg midazolam. After transseptal puncture, lidocaine (1.5 mg/kg) was administered slowly in 100 mL saline via peripheral venous access. Supplemental midazolam (1–2 mg) and fentanyl (25 μg) were given as needed.

Propofol was reserved exclusively for direct-current cardioversion, which was performed only at the end of the procedure if required. Hemodynamics, oxygen saturation, electrocardiograph, and vigilance were continuously monitored. All medications were administered by trained EP nursing staff under electrophysiologist supervision. To preserve autonomy, participants were explicitly asked during the procedure whether they wished to convert to continuous sedation. The protocol was designed to provide effective analgesia and anxiolysis while maintaining spontaneous respiration, full consciousness, and continuous communication—enabling real-time symptom monitoring and individualized titration.

### Patient-reported outcomes

Patient experience and procedural tolerability were systematically assessed using structured paper-based questionnaires at 3 predefined time points: during the procedure, 1 hour after ablation, and on the day after the procedure. All assessments were conducted under staff supervision to ensure data accuracy.

During the procedure, patients were asked to rate pain intensity during pulsed field applications, dyspnea, and procedural anxiety using numeric rating scales from 0 to 10.^13^ These assessments were completed immediately after each ablation application to capture real-time perception of physical and emotional burden.

Immediately after the final ablation, patients rated their overall satisfaction with the sedation strategy (0%–100%) and whether they would recommend it (0%–100%).

At the same time point, patients were asked to reflect on their sensory experience during the procedure. Patients were asked whether they had perceived surrounding procedural activity—such as staff communication or ambient sounds—and, if so, to rate how calming or distracting these impressions were on a 0–10 scale. Physical autonomy was assessed by documenting whether patients were able to transfer independently from the procedure table to the hospital bed.

The same core questions were repeated 1 hour and 1 day after ablation. At these time points, patients were asked to retrospectively evaluate the intensity of pain, dyspnea, and anxiety they had experienced during the ablation and whether they still felt any of these symptoms at the time of reassessment.

### Postprocedural management

Hemostasis was achieved using vascular closure devices or a figure-of-8 suture with compression. Pressure bandages were removed after 1–4 hours, depending on the closure method. Sutures were taken out the next day. Transthoracic echocardiography was routinely performed immediately after the procedure, at 1 hour, and on the first postprocedural day to exclude pericardial effusion. Oral anticoagulation was resumed 6 hours after ablation and continued for at least 2 months. Long-term anticoagulation was guided by individual thromboembolic risk according to the CHA_2_DS_2_-VA score.^14^

### Statistical analysis

Data were tested for normality using the Shapiro–Wilk test. Continuous variables are presented as mean ± standard deviation or median (quartile 1, quartile 3), as appropriate. Temporal changes in patient-reported outcomes were analyzed using paired-samples *t* tests or Wilcoxon signed-rank tests. Categorical variables are reported as counts and percentages. All analyses were performed using IBM SPSS Statistics 29.0.1.0 (IBM Corp). All tests were 2 sided; *P* < .05 was considered statistically significant. To compare outcomes between patients with BMIs of ≥30 kg/m^2^ and <30 kg/m^2^, a subgroup analysis was performed using the same statistical framework.

## Results

### Patient and procedural characteristics

14 patients (86% male; mean age 70.6 ± 9.8 years) underwent PVI using the BiB-PFA system. Paroxysmal AF was present in 43%, with a mean CHA_2_DS_2_-VA score of 2.5 ± 1.5. Cardioversion was performed in 6 cases (43%). Median procedure and left atrial dwell times were 54.5 minutes (38.3, 60.8) and 23 minutes (19.5, 34.8), respectively. The number of pulmonary vein applications was comparable across veins, with a median of 2.5 (2.0, 4.0) at the left superior pulmonary vein, 2.5 (2.0, 3.8) at the left inferior pulmonary vein, 2.5 (2.0, 4.0) at the right inferior pulmonary vein, and 2.5 (2.0, 4.0) at the right superior pulmonary vein (RSPV). Acute PVI was achieved in all patients. Posterior wall ablation was performed in 4 patients (29%) with 4.5 applications (3.8, 5.0) at 1800 V, anterior linear ablation in 2 patients (14%) with 6.0 ± 1.4 applications at 1800 V, cavotricuspid isthmus ablation in 2 patients (14%) with 5 applications at 1800 V, and superior vena cava ablation in 2 patients (14%) with 2.5 ± 0.7 applications at 1600 ± 282.8 V. No lidocaine associated proarrhythmic effect was detected (Table 1).

**Table 1:** Baseline characteristics and procedural metrics

Variable	
Gender, male, n (%)	12 (86)
Age	70.6 ± 9.8
BMI	27.7 ± 3.7
PAF	6 (42.6)
CHA_2_DS_2_-VA score	2.5 ± 1.5
NTproBNP (ng/L)	657 ± 690.1
Creatine (μmol/L)	90.7 ± 16.2
eGFR (mL/min)	72.5 ± 16.0
Procedure duration (min)	54.5 (38.3, 60.8)
Fluoroscopy time (min)	7 (5.4, 10.5)
VOLT LA dwelling (min)	23 (19.5, 34.8)
CM (mL)	40 (40, 50)
LSPV applications	2.5 (2.0, 4.0)
LSPV energy (V)	1800
LIPV applications	2.5 (2.0, 3.8)
LIPV energy (V)	1800
RIPV applications	2.5 (2.0, 4.0)
RIPV energy (V)	1800 (1800, 1800)
RSPV applications	2.5 (2.0, 4.0)
RSPV energy (V)	1800 (1800, 1800)
PW	4 (29%)
PW applications	4.5 (3.8, 5.0)
PW energy (V)	1700
AL	2 (14%)
AL applications	6.0 ± 1.4
AL energy (V)	1800
CTI	2 (14%)
CTI applications	5
CTI energy (V)	1800
SVC	2 (14%)
SVC applications	2.5 ± 0.7
SVC energy (V)	1600 ± 282.8
Electrical cardioversion	6 (42.9%)

AL = anterior linear lesion; BMI = body mass index; CM = contrast media; CTI = cavotricuspid isthmus; LA = left atrium; LIPV = left inferior pulmonary vein; LSPV = left superior pulmonary vein; NTproBNP = N-terminal pro-B-type natriuretic peptide; PAF = paroxysmal atrial fibrillation; PW = posterior wall; RIPV = right inferior pulmonary vein; RSPV = right superior pulmonary vein; SVC = superior vena cava.

### Sedation and analgesia

Sedation was administered solely by the EP team using a predefined multimodal protocol. Cumulative doses were fentanyl 116.0 ± 38.5 μg (1.5 ± 0.6 μg/kg), midazolam 4.5 ± 2.1 mg (0.05 ± 0.02 mg/kg), lidocaine 118.8 ± 30.9 mg (1.4 ± 0.2 mg/kg), and metamizole 2 g (0.02 ± 0.0 mg/kg). Propofol (86.7 ± 61.2 mg; 0.98 ± 0.63 mg/kg) was administered exclusively for cardioversion in select patients. No escalation to deep sedation was required in any case (Table 2).

**Table 2:** Drug dosages per patient

Fentanyl (μg), total Fentanyl (μg/kg/bw)	116.0 ± 38.5 1.5 ± 0.6
Midazolam (mg) Midazolam (mg/kg/bw)	4.5 ± 2.1 0.05 ± 0.02
Lidocaine (mg) Lidocaine (mg/kg/bw)	118.8 ± 30.9 1.4 ± 0.2
Metamizole (mg) Metamizole (mg/kg/bw)	2 0.02 ± 0.0
Propofol (mg) (cardioversion only) Propofol (mg/kg/bw)	86.7 ± 61.2 0.98 ± 0.63

bw = body weight.

### Patient-reported outcomes

During energy delivery, patients reported transient, moderate pain with a median score of 6.5 (4.3, 8.0), most frequently in the RSPV (57%). Dyspnea and groin pain were rated low at 0.5 (0.0, 2.0) and 1.0 (0.0, 2.0), respectively, as was procedural anxiety at 0.0 (0.0, 0.0). 79% of patients reported being aware of their surroundings, such as staff communication and procedural activity, which was generally perceived as pleasant or reassuring.

Immediately after the procedure, satisfaction with the sedation approach was rated at 90% (82.5, 100), and the likelihood of recommending the same strategy was 100% (90, 100). Patients were able to transfer from the procedure table to the stretcher or bed with minimal to moderate assistance (4.9 ± 2.0 on a 0–10 scale).

At 1 hour after the procedure, patients retrospectively rated intraprocedural pain significantly lower than during the procedure itself: 2.0 (1.0, 2.8) vs 6.5 (4.3, 8.0) (*P* = .002). A similar decline was observed for dyspnea (0.0 [0.0, 0.0] vs 0.5 [0.0, 2.0]; *P* = .655) and anxiety (0.0 [0.0, 1.0] vs 0.0 [0.0, 0.0]; *P* = .346). Satisfaction continued to be high at 95% (90, 100; *P* = .203), as did recommendation at 100% (90, 100; *P* = .168).

**Figure 1:**
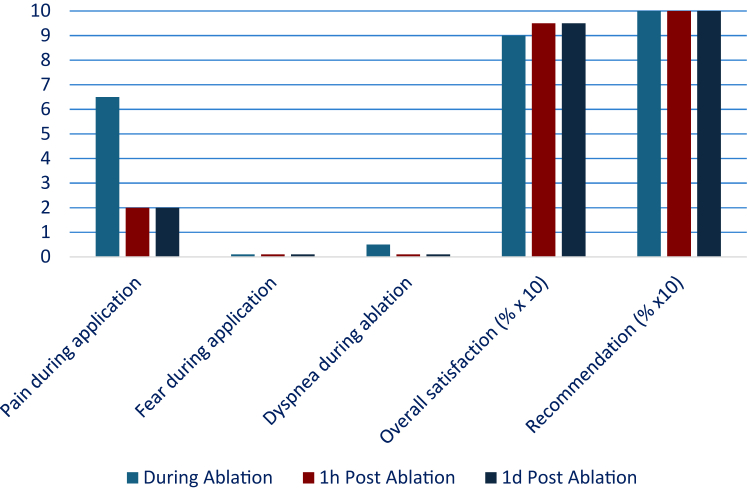
Changes in patient-reported pain, anxiety, dyspnea satisfaction, and recommendation. Values are displayed in medians.

On the day after ablation, retrospective pain perception remained low at 2.0 (1.0, 3.0; *P* = .001). No patient reported any recollection of relevant dyspnea or anxiety during the procedure at this time point. Satisfaction continued to be high at 95% (90, 100; *P* = .198), and recommendation increased slightly to 100% (92.5, 100; *P* = .048), showing a trend toward further improvement compared with immediate postprocedural ratings (Table 3, Table 4, Table 5, Table 6, Figure 1).

**Table 3:** Patient questionnaire during ablation

Pain during application (0–10)	6.5 (4.3, 8.0)
Vein with the highest pain	RSPV (57%)
Groin pain during ablation (0–10)	1.0 (0.0, 2.0)
Fear during ablation (0–10)	0.0 (0.0, 0.0)
Dyspnea during ablation (0–10)	0.5 (0.0, 2.0)
Hearing surrounding?	11 (79%)
If yes, how calming? (0–10)	6.0 (5.0, 6.0)
Overall satisfaction (0%–100%)	90 (82.5, 100)
Recommendation (0%–100%)	100 (90, 100)
Moving from the table by its own (0–10)	4.9 ± 2.0

RSPV = right superior pulmonary vein.

**Table 4:** Patient questionnaire 1 hour after ablation

Pain during application (0–10)	2.0 (1.0, 2.8)
Pain now (0–10)	0.0 (0.0, 0.0)
Groin pain during ablation (0–10)	0.0 (0.0, 1.8)
Groin pain now (0–10)	0.0 (0.0, 0.0)
Fear during ablation (0–10)	0.0 (0.0, 1.0)
Fear now (0–10)	0.0 (0.0, 0.0)
Dyspnea during ablation (0–10)	0.0 (0.0, 0.0)
Dyspnea now (0–10)	0.0 (0.0, 0.0)
Hearing surrounding?	11 (79%)
If yes, how calming? (0–10)	5.9 ± 1.2
Overall satisfaction (0%–100%)	95 (90, 100)
Recommendation (0%–100%)	100 (90, 100)

**Table 5:** Patient questionnaire 1 day after ablation

Pain during application (0–10)	2.0 (1.0, 3.0)
Pain now (0–10)	0.0 (0.0, 0.0)
Groin pain during ablation (0–10)	0.0 (0.0, 1.0)
Groin pain now (0–10)	0.0 (0.0, 0.0)
Fear during ablation (0–10)	0.0 (0.0, 0.0)
Fear now (0–10)	0.0 (0.0, 0.0)
Dyspnea during ablation (0–10)	0.0 (0.0, 0.0)
Dyspnea now (0–10)	0.0 (0.0, 0.0)
Hearing surrounding?	11 (79%)
If yes, how calming? (0–10)	6.0 (5.0, 6.5)
Overall satisfaction (0%–100%)	95 (90, 100)
Recommendation (0%–100%)	100 (92.5, 100)

**Table 6:** Patient questionnaire: comparison 1 hour vs 1 day after ablation

	Delta 1 h	*P* value	Delta 1 d	*P* value
Pain during application (0–10)	−4.5 (−8.0, −1.3)	.002	−4.5 (−5.8, −1.3)	.001
Fear during ablation (0–10)	0.0 (0.0, 0.0)	.346	0.0 (0.0, 0.0)	.773
Dyspnea during ablation (0–10)	0.0 (−0.8, 0.0)	.655	0.0 (−1.8, 0.0)	.396
Overall satisfaction (0%–100%)	0.0 (0.0, 0.75)	.203	0.0 (0.0, 0.75)	.198
Recommendation (0%–100%)	0.0 (0.0, 0.75)	.168	0.0 (0.0, 1.0)	.048

### Subgroup analysis

A BMI-based subgroup analysis (≥30 vs <30 kg/m^2^) showed no significant differences in patient-reported outcomes or sedative dose per kg body weight (Table 7).

**Table 7:** Subgroup analysis

	BMI of <30 kg/m^2^ n = 9	BMI of ≥30 kg/m^2^ n = 5	*P* value
Sedation dosage			
Fentanyl (μg/kg/bw)	1.6 ± 0.6	1.2 ± 0.4	.228
Midazolam (mg/kg/bw)	0.05 ± 0.03	0.05 ± 0.02	.892
Propofol (mg/kg/bw)	0.6 ± 0.5	1.5 ± 0.2	.103
Patient questionnaire during ablation			
Pain during application (0–10)	7.0 (5.0, 8.0)	6.0 (2.0, 6.0)	.254
Fear during ablation (0–10)	0.0 (0.0, 0.0)	0.0 (0.0, 0.0)	.589
Dyspnea during ablation (0–10)	1.0 (0.0, 2.0)	0.5 (0.0, 2.0)	.930
Overall satisfaction (0%–100%)	90 (90, 100)	90 (80, 90)	.174
Recommendation (0%–100%)	100 (90, 100)	90 (80, 90)	.313
Patient questionnaire 1 h after ablation			
Pain during application (0–10)	2.0 (1.0, 3.0)	2.0 (2.0, 2.0)	.254
Fear during ablation (0–10)	0.0 (0.0, 1.0)	0.0 (0.0, 0.0)	.589
Dyspnea during ablation (0–10)	0.0 (0.0, 0.0)	0.0 (0.0, 0.0)	.930
Overall satisfaction (0%–100%)	100 (90, 100)	90 (90, 90)	.174
Recommendation (0%–100%)	100 (100, 100)	90 (90, 100)	.313
Patient questionnaire 1 d after ablation			
Pain during application (0–10)	2.0 (1.0, 3.0)	1.0 (1.0, 2.0)	.346
Fear during ablation (0–10)	0.0 (0.0, 0.0)	0.0 (0.0, 0.0)	.423
Dyspnea during ablation (0–10)	0.0 (0.0, 0.0)	0.0 (0.0, 2.0)	.679
Overall satisfaction (0%–100%)	100 (100, 100)	90 (90, 90)	.144
Recommendation (0%–100%)	100 (100, 100)	90 (90, 100)	.275

BMI = body mass index; bw = body weight.

## Discussion

This prospective proof-of-concept study is the first to systematically evaluate the tolerability and procedural performance of PFA using the BiB-PFA system under conscious sedation without the use of deep sedation. The concept of “awake ablation” has gained increasing interest to reduce sedation-related risks and procedural complexity, yet clinical data in this setting remain limited.

The present findings support the feasibility and safety of this minimalist sedation strategy. Key observations from the study include the following:

• Awake PVI using BiB-PFA seems safe and feasible, with no need for conversion to deep sedation in any case.
• Conscious sedation combining metamizole, fentanyl, midazolam, and lidocaine effectively controlled procedural discomfort while maintaining spontaneous respiration and patient responsiveness.
• Patient-reported tolerability was high, with low median scores for pain, dyspnea, and anxiety during ablation and strong postprocedural satisfaction.
• The RSPV was most frequently associated with transient discomfort, consistent with previous ablation studies.
• Avoidance of propofol increases procedural flexibility and may reduce reliance on anesthesiology support in centers with limited resources.

In current EP practice, PVI is typically performed under deep sedation or general anesthesia to ensure procedural control. However, propofol—the most commonly used agent—is subject to regulatory restrictions in many European health care systems, where its administration is restricted to anesthesiologists or certified sedation personnel.^7^^,^^10^ This limits procedural flexibility and increases resource requirements, particularly in centers without routine anesthesiology support. Furthermore, deep sedation carries well-known clinical risks, including respiratory depression, hemodynamic instability, and delayed recovery—factors especially relevant in elderly or multimorbid patients. In response to these limitations, a conscious sedation protocol was developed for this study, aiming to maintain spontaneous ventilation and patient interaction while ensuring adequate analgesia and anxiolysis.

### Sedation strategy, pharmacologic rationale, and implementation considerations

Metamizole was selected for its peripheral analgesic efficacy and excellent hemodynamic tolerability. Fentanyl, a short-acting opioid, was used in low to moderate, titrated doses to blunt acute procedural pain. Lidocaine was administered as a short infusion immediately prior to ablation. Although IV lidocaine has not yet been systematically studied in the setting of PVI, its use was based on established evidence from visceral and vascular surgery, where perioperative administration has been shown to reduce intra- and postoperative opioid requirements and improve patient comfort without increasing complication rates.^15^^,^^16^ In the context of brief, high-intensity nociceptive stimuli during PFA, this pharmacologic rationale supported the inclusion of lidocaine as part of a multimodal analgesic strategy. Midazolam provided anxiolysis and conscious sedation. Although appropriate, its amnestic properties introduce a potential limitation in interpreting patient-reported outcomes, given that retrospective assessments of discomfort or anxiety may be affected by partial memory suppression. Although midazolam was administered in moderate doses and all patients remained responsive and able to verbally recount procedural events, partial memory suppression cannot be excluded. Therefore, retrospective reductions in perceived pain or anxiety may reflect not only true procedural tolerability but also some degree of sedation-related recall attenuation. To address this, subjective feedback was collected immediately after the procedure and again at predefined time points. Future studies incorporating formal memory testing would allow more definitive conclusions. In the study by Calvert et al,^17^ 8 patients underwent PVI using the pentaspline Farapulse system under moderate sedation without general anesthesia. The sedation regimen consisted solely of midazolam and fentanyl; no adjunctive agents such as lidocaine or nonopioid analgesics were administered. The average fentanyl dose was 209 μg, and the mean midazolam dose was 5.1 mg. In contrast, all patients in our study received a multimodal analgesic protocol. The median fentanyl dose in our cohort was 116.0 μg, and the median midazolam dose was 4.5 mg—both notably lower than in the Calvert cohort.^17^ This reduction in sedative and opioid use may reflect the synergistic analgesic effects of lidocaine and metamizole, which likely contributed to sufficient procedural comfort while preserving patient interaction and minimizing the risk of oversedation. In addition, the observed difference may in part be attributable to the catheter design itself: in contrast to the BiB-PFA system used in our study, the pentaspline catheter has been associated with more pronounced motor responses during energy delivery, potentially necessitating deeper sedation to suppress visible muscular contractions and maintain procedural control.

However, it is important to acknowledge that the successful implementation of this approach depends not only on pharmacologic selection but also on procedural experience and team coordination. The favorable outcomes observed in our center likely reflect a high degree of operator familiarity and established team routines with minimalist workflows. Communication, sedation titration, and patient feedback interpretation all require dedicated attention and internal consistency. Thus, the awake workflow should be viewed not merely as a reduction in sedation, but as a distinct procedural paradigm—one that benefits from structured implementation and cumulative team experience.

### Patient comfort, pain perception, and satisfaction

Pain, anxiety, and dyspnea are determinants of procedural acceptance in conscious ablation workflows. In our cohort, patients occasionally reported brief, moderate pain during energy delivery—most commonly in the RSPV. These sensations were short lived and did not lead to persistent discomfort, respiratory distress, or heightened anxiety. Dyspnea was rarely reported, and overall stress levels remained low. Despite transient discomfort, most patients described the procedure as well tolerated, with a strong sense of safety and control. They frequently emphasized the value of remaining communicative and aware, and almost all stated that they would choose the same sedation strategy again and recommend it to others.

These findings are consistent with the observations by Calvert et al,^17^ who also noted procedural pain under moderate sedation but high patient acceptance and willingness to recommend the approach. What sets our study apart is the structured, time-resolved assessment of patient experience during and after the procedure. By capturing real-time intraprocedural feedback and follow-up assessments at 1 hour and 1 day after ablation, we documented a consistent decline in the subjective intensity and perceived relevance of discomfort. Therefore, it is plausible that the observed attenuation of discomfort reflects a combined effect of the ultrashort PFA impulses delivered by the BiB-PFA system and the pharmacologic properties of midazolam.

Furthermore, BMI-stratified analysis (≥30 kg/m^2^ vs <30 kg/m^2^) showed no significant differences in patient-reported tolerance or drug dosage adapted to body weight. Although exploratory, this suggests that awake BiB-PFA may be feasible across a broad range of body habitus. However, larger prospective studies are required to confirm these findings.

Patient satisfaction remained high across all time points, and the near-universal willingness to recommend the awake approach underscores its clinical viability and acceptability.

These observations demonstrate the feasibility and preliminary safety of a structured, EP-managed awake workflow for BiB-PFA in this cohort. Although these initial results are encouraging, validation in larger and more diverse patient populations is required before broad applicability can be confirmed. A prospective randomized study comparing conscious with deep sedation workflows is planned to further evaluate safety, tolerability, and generalizability.

### Limitations

It was a single-center, nonrandomized observational study, which limits generalizability. The sample size was modest and sufficient for assessing procedural tolerance and sedation strategy but underpowered to detect rare events. This study did not include a direct comparison with deep sedation workflows. The prospective randomized study comparing both approaches will be performed to further confirm these findings. Patient experience was evaluated using simple numeric scales, which, although practical intraprocedurally, may not fully capture discomfort or anxiety. More detailed instruments could offer additional insights in future studies. High tolerability may reflect BiB-specific features, but, without direct comparison with other PFA systems, catheter-related effects remain speculative. Favorable outcomes may in part result from operator and team expertise, given that centers less familiar with awake workflows may encounter a learning curve in communication, sedation titration, and expectation management in the absence of anesthesiology support. Finally, because no dedicated memory assessment was performed, the possibility of an amnestic influence of midazolam on retrospective pain reporting cannot be ruled out.

## Conclusion

This study demonstrates that PVI using a balloon-based PFA system is feasible, safe, and well tolerated under a nonpropofol, electrophysiologist-managed sedation protocol. The approach maintained procedural control and lesion consistency while reducing sedation-related burden, with high patient satisfaction and willingness to recommend this approach. These findings support the feasibility of an awake workflow in this setting and highlight the need for confirmation in larger cohorts for generalizability. A prospective randomized study comparing conscious and deep sedation strategies is planned to validate these results and further guide sedation approaches in BiB-PFA.

## Declaration of generative AI and AI-assisted technologies in the writing process

During the preparation of this work, the authors used ChatGPT to improve language. After using this tool/service, the authors reviewed and edited the content as needed and take full responsibility for the content of the publication.

## Disclosures

R.T. has received honoraria for lectures from Pfizer, Abbott, Biosense Webster, Boston Scientific, Doctrina Med, cme4u, Medtronic, Radcliff Cardiology, and Wikonect. He has received honoraria for advisory board participation and consulting from Boston Scientific, Biosense Webster, Capvision, Guidepoint, Haemonetics, Medtronic, Philips, and Abbott. His institution has received research funding or participated in clinical trials sponsored by Biotronik, Abbott, Boston Scientific, Medtronic, Lifetech, and Johnson & Johnson. He has also received travel grants from Biosense Webster, Abbott, Boston Scientific, Medtronic, and Philips. J.P.W. received funding from the German Foundation of Heart Research (F/29/19), speaker fees from Abbott and Doctrina Med, and travel grants from Boston Scientific in each case unrelated to this project. C.E. received travel grants and research grants from Abbott, Boston Scientific, Lifetech, Biosense Webster, and CardioFocus and speaker honoraria from Abbott, Boston Scientific, Biosense Webster, CardioFocus, C.T.I. GmbH and Doctrina Med. S.S.P. has received travel grants and congress grants from Lifetech and educational grants and a speaker grant from Abbott Medical. He is a medical consultant by Active Health. J.N. has received honoraria for lectures from Abbott. K.H.K. reports grants and personal fees from Abbott Vascular, Medtronic, and Biosense Webster outside the submitted work. S.H. has received travel and education grants from Abbott.

## References

[1] Chun K.-R.J., Miklavčič D., Vlachos K. (2024). State-of-the-art pulsed field ablation for cardiac arrhythmias: ongoing evolution and future perspective. Europace.

[2] Schmidt B., Bordignon S., Neven K. (2023). EUropean real-world outcomes with Pulsed field ablatiOn in patients with symptomatic atRIAl fibrillation: lessons from the multi-centre EU-PORIA registry. Europace.

[3] Ekanem E., Neuzil P., Reichlin T. (2024). Safety of pulsed field ablation in more than 17,000 patients with atrial fibrillation in the MANIFEST-17K study. Nat Med.

[4] Boersma L., Andrade J.G., Betts T. (2023). Progress in atrial fibrillation ablation during 25 years of Europace journal. Europace.

[5] Tilz R.R., Chierchia G.B., Gunawardene M. (2025). Safety and effectiveness of the first balloon-in-basket pulsed field ablation system for the treatment of atrial fibrillation: VOLT CE Mark Study 6-month results. Europace.

[6] Sanders P., Healy S., Emami M., Kotschet E., Miller A., Kalman J.M. (2024). Initial clinical experience with the balloon-in-basket pulsed field ablation system: acute results of the VOLT CE mark feasibility study. Europace.

[7] Schmidt B., Bordignon S., Tohoku S. (2022). 5S Study: safe and simple single shot pulmonary vein isolation with pulsed field ablation using sedation. Circ Arrhythm Electrophysiol.

[8] Riis-Vestergaard L.D., Tønnesen J., Ruwald M.H. (2024). General anaesthesia compared to conscious sedation for first-time atrial fibrillation catheter ablation-a Danish nationwide cohort study. Europace.

[9] Tilz R.R., Busch S., Chun K.R.J. (2024). Analgosedierung in der Kardiologie. Konsensuspapier der DGK und DGAI 2024. Kardiologie.

[10] Garcia R., Waldmann V., Vanduynhoven P. (2021). Worldwide sedation strategies for atrial fibrillation ablation: current status and evolution over the last decade. Europace.

[11] Buia V., Bastian D., Stangl D. (2025). Predictors of hemodynamic and respiratory instability during deep sedation for left atrial ablation: preliminary results from a retrospective german cohort. Europace.

[12] World Medical Association (2025). World Medical Association Declaration of Helsinki: Ethical Principles for Medical Research Involving Human Participants. JAMA.

[13] Ferreira-Valente M.A., Pais-Ribeiro J.L., Jensen M.P. (2011). Validity of four pain intensity rating scales. Pain.

[14] Van Gelder I.C., Rienstra M., Bunting K.V. (2024). 2024 ESC Guidelines for the management of atrial fibrillation developed in collaboration with the European Association for Cardio-Thoracic Surgery (EACTS). Eur Heart J.

[15] Weibel S., Jokinen J., Pace N.L. (2016). Efficacy and safety of intravenous lidocaine for postoperative analgesia and recovery after surgery: a systematic review with trial sequential analysis. Br J Anaesth.

[16] Gajniak D., Mendrala K., Cyzowski T., Polak M., Gierek D., Krzych Ł.J. (2023). Efficacy of Lidocaine Infusion in High-Risk Vascular Surgery-A Randomized, Double-Blind, Placebo-Controlled Single-Center Clinical Trial. J Clin Med.

[17] Calvert P., Mills M.T., Murray B. (2025). Feasibility of pulsed field ablation for atrial fibrillation under mild conscious sedation. J Interv Card Electrophysiol.

